# Homoharringtonine enhances cytarabine-induced apoptosis in acute myeloid leukaemia by regulating the p38 MAPK/H2AX/Mcl-1 axis

**DOI:** 10.1186/s12885-024-12286-7

**Published:** 2024-04-24

**Authors:** Yang Qiu, Lu Bai, Haosen Zhao, Xifan Mei

**Affiliations:** 1 School of Pharmacy, Jinzhou Medical University, Jinzhou, 121001 Liaoning China; 2 Jinzhou Medical University, Jinzhou, 121001 Liaoning China; 3 Liaoning Provincial Collaborative Innovation Center for Medical Testing and Drug Research, Jinzhou Medical University, Jinzhou, 121001 Liaoning China; 4 Liaoning Provincial Key Laboratory of Marine Bioactive Substances, Jinzhou Medical University, Jinzhou, 121001 Liaoning China; 5 Technological Innovation Center of Liaoning Pharmaceutical Action and Quality Evaluation, Jinzhou Medical University, Jinzhou, 121001 Liaoning China; 6 Affiliated Third Hospital of Jinzhou Medical University, Jinzhou, 121001 Liaoning China

**Keywords:** AML, Homoharringtonine, Cytarabine, p38 MAPK, Apoptosis

## Abstract

Acute myeloid leukaemia (AML) is a fatal haematopoietic malignancy and is treated with the conventional combination of cytarabine (Ara-C) and daunorubicin (Dau). The survival rate of AML patients is lower due to the cardiotoxicity of daunorubicin. Clinically, homoharringtonine (HHT) plus Ara-C has been reported to be equally effective as Dau plus Ara-C in some types of AML patients with less toxic effects. We utilized the clinical use of homoharringtonine in combination with Ara-C to test its combination mechanism. We found that the insensitivity of AML cells to cytarabine-induced apoptosis is associated with increased Mcl-1 stability and p38 inactivation. HHT downregulates Mcl-1, phosphorylates H2AX and induces apoptosis by activating p38 MAPK. Inactivation of p38 through inhibitors and siRNA blocks apoptosis, H2AX phosphorylation and Mcl-1 reduction. HHT enhances Ara-C activation of the p38 MAPK signalling pathway, overcoming Ara-C tolerance to cell apoptosis by regulating the p38/H2AX/Mcl-1 axis. The optimal ratio of HHT to Ara-C for synergistic lethality in AML cells is 1:4 (M/M). HHT synergistically induces apoptosis in combination with Ara-C in vitro and prolongs the survival of xenografts. We provide a new mechanism for AML treatment by regulating the p38 MAPK/H2AX/Mcl-1 axis to improve cytarabine therapy.

**Supplementary Information:**

The online version contains supplementary material available at 10.1186/s12885-024-12286-7.

## Introduction

Acute myeloid leukaemia (AML) is one of the deadliest myeloid haematopoietic malignancies and is characterized mainly by uncontrolled proliferation of blood cells from the myeloid lineage [1, 2]. 7 + 3 therapy, the standard treatment for AML patients, involves cytarabine (Ara-C) and daunorubicin (Dau) [3]. Only 30 to 40% of adult AML patients and 5 to 15% of elderly AML patients survive more than 5 years [4]. Most patients treated with standard therapy die from the disease due to relapse and toxicity of the treatment, leading to shorter survival in AML patients [5]. Ara-C alone was used for elderly AML patients, and new combinations with less toxicity have been developed. Significant progress has been made in the treatment of AML with the development of small molecular inhibitors, such as the FLT3 inhibitor midostaurin, the Bcl-2 inhibitor venetoclax and IDH inhibitors [6–8]. These approved drugs should be integrated into combination regimens with chemotherapy for the management of AML. The addition of HHT significantly improves traditional treatment regimens and reduces cardiac toxicity caused by doxorubicin in AML patients [9, 10]. Clinically, HHT, which has relatively low extramedullary and myocardial toxicity, was combined with Ara-C to treat elderly patients with AML [11]. Although HHT plus Ara-C had a less toxic effect than Dau plus Ara-C, the synergistic mechanism of this combination is worthy of further study.

Cell apoptosis evasion is the main cause of leukaemia occurrence and chemotherapy failure. Apoptosis is regulated by antiapoptotic proteins (Mcl-1, Bcl-2, and Bcl-xL), proapoptotic proteins (Bax and Bak) and BH3-only proteins (Bim, PUMA, Noxa, Bad, and Bid). The Mcl-1 protein plays a more important role than the Bcl-2 protein in the development of leukaemia [12]. Mcl-1 is considered a key target for AML treatment [13]. Ara-C stabilized the protein levels of Mcl-1 in AML cells without PARP cleavage. Mcl-1 blocks cell apoptosis and DNA damage [14]. Reducing the Mcl-1 protein in AML cells could be an effective way to improve cytarabine-based combination therapy.

HHT is a natural alkaloid obtained from *Cephalotaxus* species that is widely used in China for AML patients. HHT can inhibit the proliferation of leukaemia cells and induce apoptosis or differentiation [15–17]. HHT rapidly downregulates Mcl-1 to induce cell apoptosis through protein synthesis inhibition [18]. In cells that are less dependent on pro-apoptotic proteins, HHT can also cause cell death, indicating alternative mechanisms of function. Translation inhibitors have been confirmed to cause cell death via multiple mechanisms [19]. We hypothesize that HHT reduces the Mcl-1 protein level and accelerates Ara-C-induced leukaemia cell apoptosis through multiple pathways.

MAPK signalling pathways, including the JNK, p38 and ERK MAPK pathways, are involved in cell apoptosis through various pathways [20]. p38 and JNK phosphorylation are usually pro-apoptosis, and ERK phosphorylation is usually anti-apoptosis. Activation of the p38/JNK MAPK pathway affects downstream targets and accelerates cytochrome c release and caspase activation [21]. p38 MAPK induces cell apoptosis by regulating H2AX phosphorylation, as the proteins γ-H2AX and p38 MAPK are located in apoptotic bodies [22, 23]. H2AX phosphorylation is a marker protein for DNA damage and can induce cell apoptosis [24]. Moreover, p38 MAPK activation has been reported to reduce Mcl-1 expression [25]. We hypothesize that the combination of HHT and Ara-C induces cell apoptosis via MAPK, DNA damage, and the anti-Mcl-1 protein.

In this study, we selected four AML cell lines to test their ability to inhibit the growth of HHT and Ara-C and analysed their ability to induce apoptosis. Using sensitive HL-60 cells and insensitive THP-1 cells, we found that the ability of cytarabine to promote AML cell apoptosis is associated with Mcl-1 stability and p38 inactivation. HHT activates p38/H2AX, reduces the Mcl-1 protein, promotes Ara-C-mediated apoptosis in vitro, and prolongs the survival of THP-1 xenografts. In this manuscript, we tested the ability of the clinical use of HHT, an antileukaemia drug, to accelerate the ability of Ara-C to induce cell apoptosis through the p38/H2AX/Mcl-1 axis.

## Materials and methods

### Reagents

HHT for injection was purchased from Minsheng Pharmaceutical Group (Hangzhou, China). Cytarabine and PH797804 were obtained from Selleck Chemicals (Houston, TX, USA). SB203580 was acquired from Sigma–Aldrich, Inc. Antibodies against PARP and caspase-3 were purchased from BD Biosciences (Maryland, USA); antibodies against cleaved caspase-3 (C-C3), p-p38, p-JNK, ERK, p-ERK and p-H2AX were obtained from Cell Signaling Technology, Inc. (Beverly, MA); and antibodies against Mcl-1, p38α, JNK1 and β-actin were acquired from Santa Cruz Biotechnology (San Diego, CA).

### Cell lines

HL-60, MOLM-13, U937 and THP-1 cells were obtained from American Type Culture Collection (ATCC) (Rockville, MD). The cells were cultured in RPMI 1640 medium supplemented with 100 U/ml penicillin, 100 μg/ml streptomycin, 1 mM L-glutamine and 10% (v/v) heat-inactivated foetal bovine serum.

### Quantification of apoptotic cells

#### Detection of apoptosis by acridine orange/ethidium bromide (AO-EB) staining

AML cells were seeded and incubated at a density of 1 × 10^5^ in 24-well plates. After treatment with different drugs for 24 h, the cells were collected by centrifugation and washed once with PBS. The dye mixture of 100 μg/ml AO and 100 μg/ml EB was diluted in PBS, mixed with the cell suspension, and placed onto microscope slides [26]. Three hundred cells were observed and counted by fluorescence microscopy. The percentage of apoptotic cells was calculated. The data are presented as the mean inhibition rates±SD from at least three independent experiments.

#### Detection of apoptosis by Annexin V/PI staining

AML cells were seeded and incubated at a density of 1 × 10^5^ in 6-well plates. The cells treated with different drugs were collected by centrifugation and washed once with PBS. After removing the supernatant, the precipitate was mixed with binding buffer, and then Annexin V and PI dyes were added in proportion according to the instructions of the Annexin V-FITC apoptosis kit. A total of 10,000 cells were collected by flow cytometry, and the percentage of apoptotic cells was calculated.

More details on the apoptosis experiments were provided in the relevant literature [14].

### Cell survival and inhibition rates

AML cells were seeded and incubated at a density of 1 × 10^5^ in 24-well plates. After treatment with different drugs for 24 h, the trypan blue working solution and cell suspension were mixed at a 1:1 (v/v) ratio. Trypan blue dye was used to stain the damaged cells, and the cells were counted under a microscope using a counting plate. The total number of cells and the number of trypan blue-stained cells were recorded. The cell survival rate (%) = number of surviving cells/total number of cells× 100%. The inhibition rate (%) = (1- total number of cells in the dosing group/total number of cells in the blank group) × 100%. The Bliss method was used to calculate the concentration of half cell growth inhibition (GI_50_).

### Statistical analysis of the combination effect

The combination index (CI) was measured to determine whether the combination drugs were synergistic, additive or antagonistic. CompuSyn software was used to calculate CIs. A CI < 0.9 indicated synergistic effects, 0.9 < CI < 1.1 indicated additive effects, and CI > 1.1 indicated antagonistic effects.

### Western blot analysis

AML cells were seeded and incubated at a density of 1 × 10^5^ in 6-well plates. After treatment with different drugs for 24 h, the treated cells were collected by centrifugation and washed once with PBS. After removing the supernatant, the cells were lysed with NP-40 lysis buffer containing proteinase inhibitors. The protein concentration was quantified by a BCA protein quantitative kit. Protein samples (40 μg) were separated by 10% SDS–PAGE and then transferred to cellulose nitrate (NC) membranes by wet rotation. The NC membranes were stained with 0.2% Ponceau and subsequently sealed with TBST containing 5% skim milk. After 2 hours at room temperature, the sections were incubated with specific antibodies against the target protein and NC membrane overnight at 4 °C. The next day, the strips were cleaned and then incubated at room temperature for 1 hour with the secondary antibody. After the strips were removed, an enhanced chemiluminescence kit was used for chemiluminescence, and X-ray film was added for development. More details were provided in our previous reports [17].

### SiRNA

A total of 10^6^ THP-1 cells were collected. According to the instructions of the Lonza Transfection Kit, 100 μL of mixed electroconversion working solution and 400 nM siRNA were transferred to resuspended cell cakes, which were placed in an electroconversion cup for transfection. The cells were transferred to culture media and incubated at 37 °C for 24 h before they were harvested for drug treatment.

### Xenografts

NOD-SCID mice were purchased from Beijing Weitonglihua and raised at the SPF level animal experimental centre of Jinzhou Medical University. The experimental procedure followed animal protection regulations and guidelines. Twenty mice were inoculated with 5 × 10^6^ THP-1 cells subcutaneously into the lateral armpits. The tumour volume reached 100 mm^3^, and 4 groups of randomly assigned mice were established: control, Ara-C (50 mg/kg), HHT (1 mg/kg), and Ara-C plus HHT groups. HHT and/or Ara-C were administered intraperitoneally once a day for 7 consecutive days. After the mice were euthanized via the carotid artery, the tumours were weighed and compared.

In another experiment, 20 mice were injected with cyclophosphamide for 2 days (150 mg/kg, i.p.) and inoculated with 5 × 10^6^ THP-1 cells through the tail vein. Four groups of randomly assigned mice were established: control, Ara-C (50 mg/kg), HHT (1 mg/kg), and Ara-C plus HHT groups. Ara-C was administered intraperitoneally once a day for 7 consecutive days. HHT was administered intraperitoneally once a day for 14 consecutive days. The body weights and survival times of the mice were recorded. The median survival and the increase in lifespan (ILS) were calculated.

## Statistical analysis

The data were analysed by using SPSS, and the statistical significance was assessed by analysis of variance (ANOVA). A value of *P* < 0.05 indicated a statistically significant difference (*). A value of *P* < 0.01 was considered to indicate a statistically significant difference (**). A value of *P* < 0.001 was considered to indicate a statistically significant difference (***).

## Results

### Insensitivity to cytarabine-induced apoptosis is associated with Mcl-1 stability and p38 inactivation

**Fig. 1:**
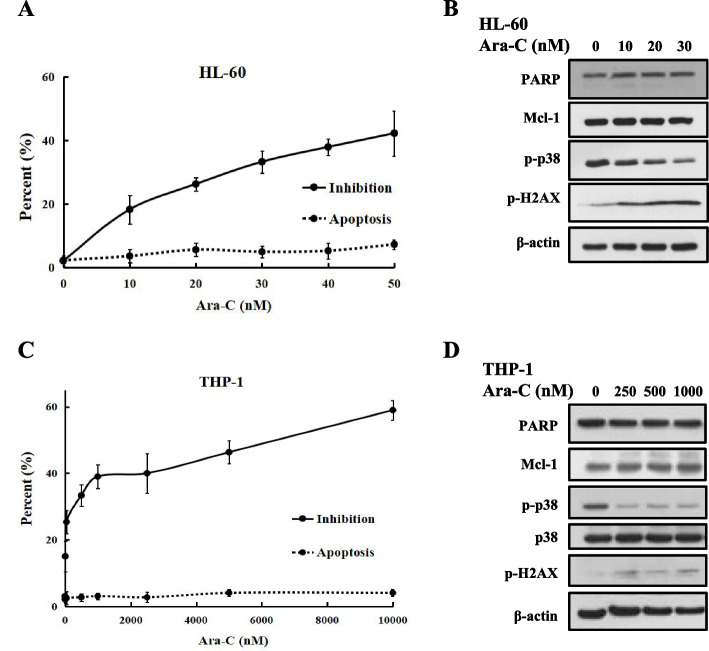
Mcl-1 levels influenced cytarabine-induced apoptosis in AML cells. **A**, **C**, The cell inhibition and apoptosis percentages of HL-60 or THP-1 cells treated with increasing concentrations of Ara-C for 24 h were examined by trypan blue and AO-EB staining. **B**, **D**, Western blot analysis of PARP, p-p38, Mcl-1 and p-H2AX protein expression in HL-60 or THP-1 cells treated with Ara-C for 24 h

Clinically, Ara-C alone was used for elderly patients with AML. We analysed cell inhibition in AML cells based on trypan blue staining at the indicated concentrations of Ara-C. The HL-60 and U937 cells were sensitive to Ara-C at concentrations of 75 nM and 40 nM, which inhibited 50.3 and 54.8%, respectively, of the cell growth. The THP-1 cells were insensitive to Ara-C, with a GI_50_ > 10 μM (Fig. S1A). We selected two cell lines, namely, sensitive HL-60 cells and insensitive THP-1 cells, to analyse apoptosis based on morphological changes after treatment with Ara-C and AO/EB. Ara-C at 50 nM and 10 μM inhibited 42.3 and 59.0% of the cell growth, respectively, and only induced apoptosis in 7.3 and 4.0% of the HL-60 and THP-1 cells, respectively (Fig. 1A, C). The levels of Mcl-1 influence cell sensitivity to Ara-C-induced apoptosis and DNA damage [14]. Mcl-1 is regulated by the upstream (p38, JNK, and ERK) MAPK kinase pathway [27]. We tested Mcl-1 and the DNA damage markers γ-H2AX and p-p38 in HL-60 and THP-1 cells treated with Ara-*C. *Ara-C decreased the levels of p-p38, weakly increased p-H2AX, and maintained Mcl-1 levels without PARP cleavage (Fig. 1B, D). Insensitivity to apoptosis and DNA damage caused by cytarabine is associated with Mcl-1 stability and p38 inactivation.

### HHT-induced apoptosis is associated with p38 activation, DNA damage, and Mcl-1 downregulation

**Fig. 2:**
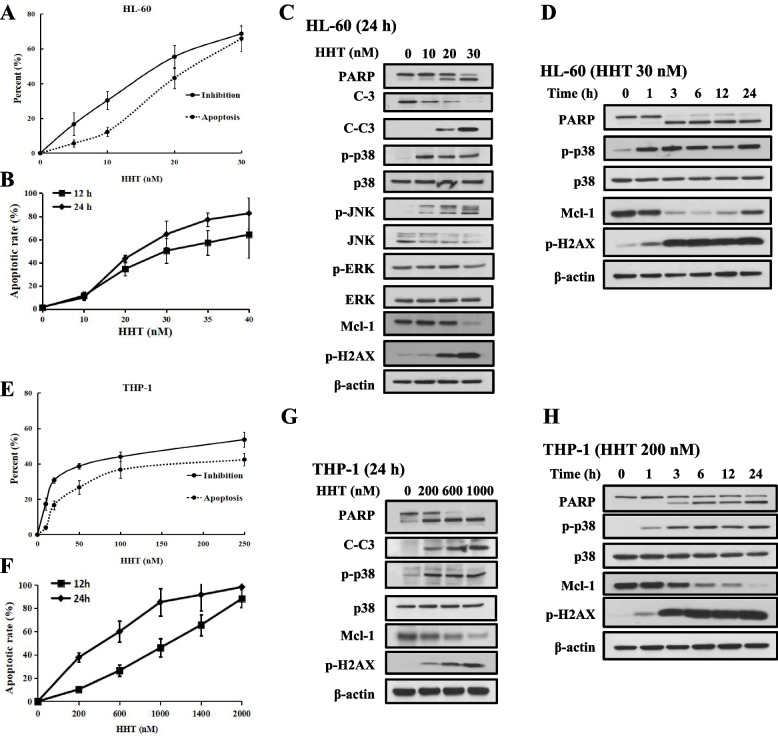
HHT induced apoptosis, decreased the protein levels of Mcl-1, and increased the phosphorylation of p38 and H2AX in AML cells. **A**, **E**, The cell inhibition and apoptosis percentages of HL-60 or THP-1 cells treated with increasing concentrations of HHT for 24 h were examined by trypan blue and AO-EB staining. **B**, **F**, The percentage of HL-60 or THP-1 cells in which apoptosis was induced, respectively, after treatment with increasing concentrations of HHT for 12 h or 24 h was examined by AO-EB staining. **C**, **G**, Western blot analysis of the protein expression of PARP, caspase-3, cleaved caspase-3 (C-C3), MAPK kinase, Mcl-1 and p-H2AX in HL-60 and THP-1 cells treated with increasing concentrations of HHT for 24 h. **D**, **H**, Protein expression in cells treated with HHT for 1, 3, 6, 12 or 24 h

HL-60, MOLM-13, U937 and THP-1 cells were sensitive to HHT, and the GI_50_ ranged from 10 to 200 nM (Fig. S2A). The apoptosis and growth inhibition induced by HHT were measured in the HL-60 and THP-1 cell lines after 24 h. HHT at 30 nM and 250 μM inhibited 68.7 and 53.7% of the cell growth, respectively, and induced apoptosis in 65.9 and 42.3% of the HL-60 and THP-1 cells, respectively (Fig. 2A, E). The inhibitory effect of HHT on cell growth was related to its ability to induce cell apoptosis. We tested HL-60 cells treated with low concentrations (10–40 nM) of HHT for 24 h. HHT at a concentration of 30 nM for 12 h induced 50.4% of HL-60 cells to undergo apoptosis, which increased to 64.6% within 24 h (Fig. 2B). Compared with HL-60 cells, THP-1 cells were less sensitive to HHT-induced apoptosis. HHT at a concentration of 600 nM for 12 h induced apoptosis in 26.3% of the THP-1 cells and increased apoptosis in 59.9% of the cells after 24 h (Fig. 2F). The results indicated that HHT induced the apoptosis of AML cells in a time- and concentration-dependent manner. HHT induces AML cell apoptosis via Mcl-1 downregulation and is an FDA-approved treatment for clinical chronic myeloid leukaemia [28, 29]. We analysed the levels of Mcl-1, p-H2AX, and upstream MAPK kinase proteins in HL-60 and THP-1 cells treated with HHT for 24 h. In HL-60 cells, 10 nM HHT activated p38 and JNK without caspase-3 cleaved, but decreased p-ERK and Mcl-1 at 30 nM (Fig. 2C). Apoptosis induction increased in response to 20–30 nM HHT, as indicated by Mcl-1 downregulation and H2AX phosphorylation (Fig. 2C), but there was no induction of the apoptotic proteins Bcl-2 and Bim [30] (Fig. S2B). After HHT treatment for 1 h, the HHT-mediated phosphorylation of p38 and H2AX increased without Mcl-1 downregulation and caspase-3 cleavage (Fig. 2D). Similar to the results in HL-60 cells, HHT activated p38 and H2AX before Mcl-1 downregulation and apoptosis induction in THP-1 cells (Fig. 2G, H). The ability of HHT to induce apoptosis was positively correlated with p-p38 and p-H2AX levels and negatively correlated with Mcl-1 levels.

### The p38 MAPK/H2AX/Mcl-1 axis plays a critical regulatory role in HHT-induced apoptosis

**Fig. 3:**
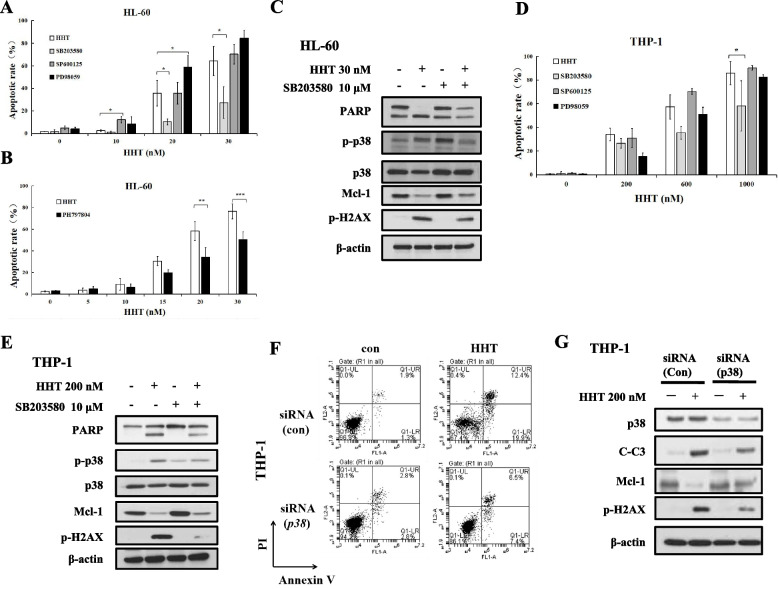
HHT induced apoptosis by p38 MAPK/H2AX/Mcl-1 axis. **A**, **B**, **D** HL-60 and THP-1 cells were pretreated with 10 μM SP600125, a p38 inhibitor (10 μM SB203580) or PH797804, an ERK inhibitor (10 μM PD98059), for 4 h and then treated with increasing concentrations of HHT for another 24 h, after which apoptosis was detected by AO-EB staining. **C**, **E**, Protein expression in HL-60 or THP-1 cells treated with SB203580 and HHT for 24 h. **F**, **G**, p38 was silenced with *p38* siRNA in THP-1 cells that were subsequently treated with HHT. *, *P* < 0.05; **, *P* < 0.01; ***, *P* < 0.001

We then tested the role of p38 MAPK in the induction of apoptosis via AO/EB staining using two p38 inhibitors, SB203580 and PH797804, which decreased the induction of apoptosis by HHT in both HL-60 and THP-1 cells (Fig. 3A, B, D). PD98059 weakly increased HHT-induced apoptosis in HL-60 cells but not in THP-1 cells. The effect of the JNK inhibitor SP600125 on The effect of HHT on apoptosis remained unchanged in both cell lines (Fig. 3A, D). SB203580 reversed PARP cleavage and reduced Mcl-1 downregulation and H2AX phosphorylation by HHT in HL-60 and THP-1 cells (Fig. 3C, E). To test the role of p38 MAPK in apoptosis induction, we used *p38* siRNA. Silencing *p38* attenuated HHT-induced apoptosis in THP-1 cells, as shown by Annexin V/PI staining (Fig. 3F). *p38* siRNA reduced the cleavage of caspase-3 and blocked Mcl-1 downregulation and H2AX activation (Fig. 3G). These results indicated that p38 MAPK can regulate Mcl-1 and DNA damage and that the p38 MAPK/H2AX/Mcl-1 axis plays a critical regulatory role in the HHT-induced apoptosis of AML cells (Fig. 3G).

### HHT plus Ara-C could synergistically induce apoptosis

**Fig. 4:**
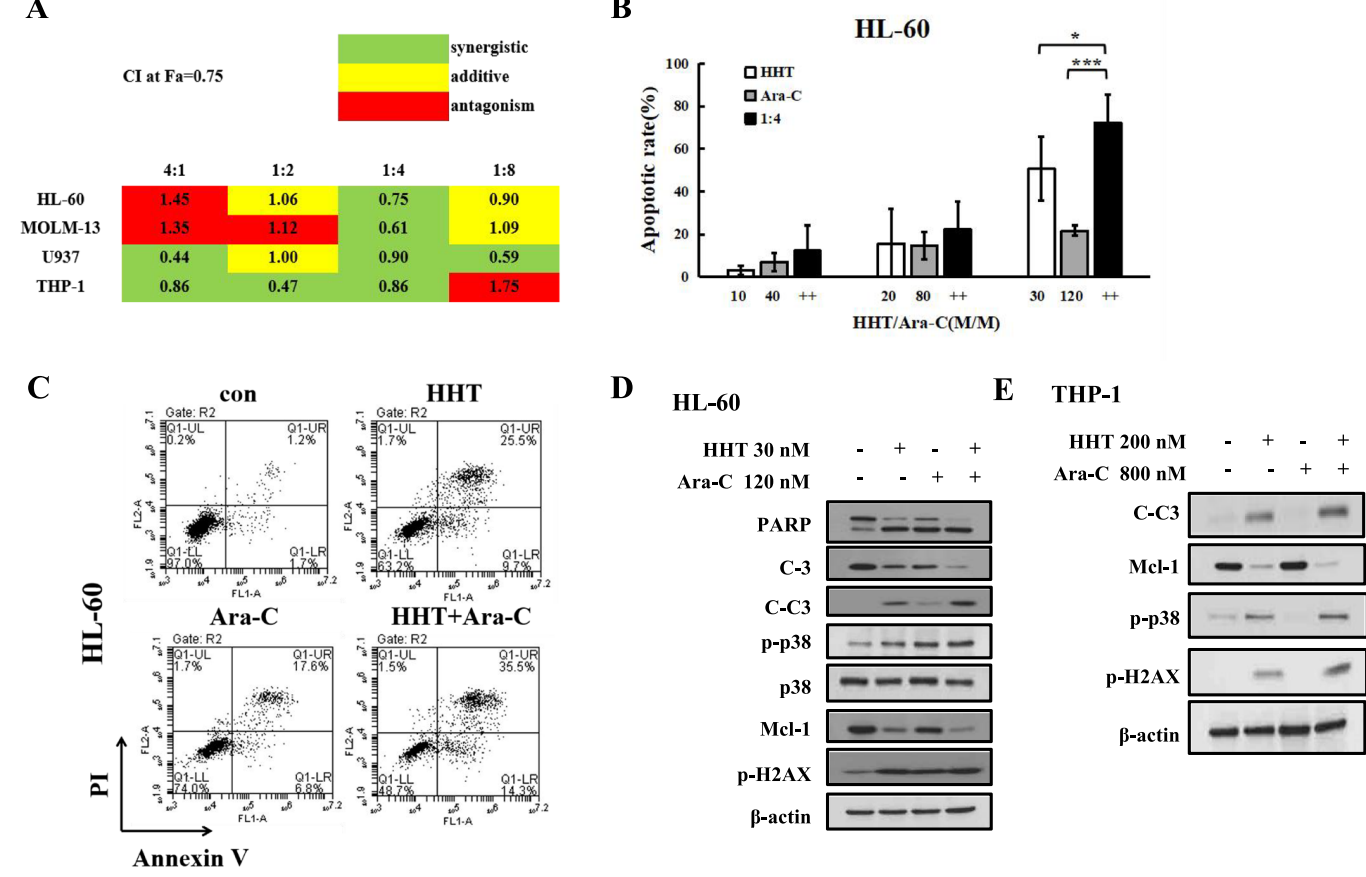
HHT plus Ara-C induced synthetic lethality in AML. **A**, CI at the ED_75_ (Fa = 0.75) of HHT plus Ara-C at different doses of 4:1, 1:2, 1:4 and 1:8 (M/M) for 24 h in HL-60, THP-1, MOLM-13 and U937 cells, where CI values < 0.9, 0.9–1.1 and > 1.1 indicate synergism, additive and antagonism, respectively. **B**, HL-60 cells treated with the combination of 10 nM, 20 nM, or 30 nM HHT and 40 nM, 80 nM, or 120 nM Ara-C at a fixed ratio of 1:4 for 24 h were examined by AO-EB staining. *, *P* < 0.05; ***, *P* < 0.001. **C**, The apoptosis of HL-60 cells treated with the combination of 30 nM HHT and 120 nM Ara-C at a fixed ratio of 1:4 for 24 h was determined by Annexin V/PI staining. **D**, **E**, Protein regulation of HL-60 and THP-1 cells treated with HHT plus Ara-C for 24 h

The growth inhibition effects of HHT and Ara-C were tested in four AML cell lines (HL-60, THP-1, MOLM-13 and U937) (Fig. S1A, S2A). The GI_50_ of HHT ranged from 13.8 nM to 213.2 nM, and that of Ara-C ranged from 47.5 nM to 7155.1 nM (Fig. S3A). HHT showed greater sensitivity in all cell lines than Ara-C. HL-60 cells were sensitive to both HHT and Ara-C, but THP-1 cells were not sensitive to HHT or Ara-C. A suitable combination ratio and drug dosage were needed [31, 32]. Based on the sensitivity of the two drugs, we selected HHT and Ara-C at different concentrations and ratios of 4:1, 1:2, 1:4 and 1:8 (M/M) for 24 h. We calculated the CI of each combination by the median effect method using CompuSyn software [33, 34]. The CIs for ED_75_ were less than 0.9 in all the AML cell lines, demonstrating synergy at a 1:4 ratio of HHT to Ara-C (Fig. 4A). We compared the effects of HHT, Ara-C, and HHT plus Ara-C at different concentrations at a 1:4 ratio (M/M). Treatment with 30 nM HHT plus 120 nM Ara-C for 24 h increased the percentage of HL-60 and MOLM-13 cells by 72.3 and 57.0%, respectively (Fig. 4B, Fig. S3B). Synergy-induced apoptosis in HL-60 cells treated with 30 nM HHT and 120 nM Ara-C was confirmed via Annexin V/PI staining (Fig. 4C). HHT in combination with Ara-C augmented the cleavage of PARP and caspase-3, increased p-H2AX and p-p38 levels and downregulated Mcl-1 at the protein level in HL-60 cells (Fig. 4D). At high concentrations, Ara-C can induce apoptosis in AML cells [35]. In HL-60 cells, Ara-C induced apoptosis in a time- and concentration-dependent manner at 25–600 nM for 12 h or 24 h (Fig. S3C) but at concentrations > 50 μM in THP-1 cells [14]. Ara-C at 120 nM induced the cleavage of PARP and caspase-3 and increased p-H2AX and p-p38 but maintained Mcl-1 levels in HL-60 cells (Fig. 4D). In THP-1 cells, 800 nM Ara-C weakly reduced p38 phosphorylation without affecting caspase-3 cleavage, Mcl-1 or p-H2AX levels (Fig. 4E). HHT not only significantly increased PARP and caspase-3 cleavage, p38 and H2AX activation, but also decreased Mcl-1 expression, which was increased by Ara-C (Fig. 4E). These data suggested that HHT enhanced Ara-C-induced apoptosis in AML cells and was associated with the p38 MAPK/H2AX/Mcl-1 axis.

### HHT enhanced Ara-C-induced apoptosis via the p38 MAPK/H2AX/Mcl-1 axis

**Fig. 5:**
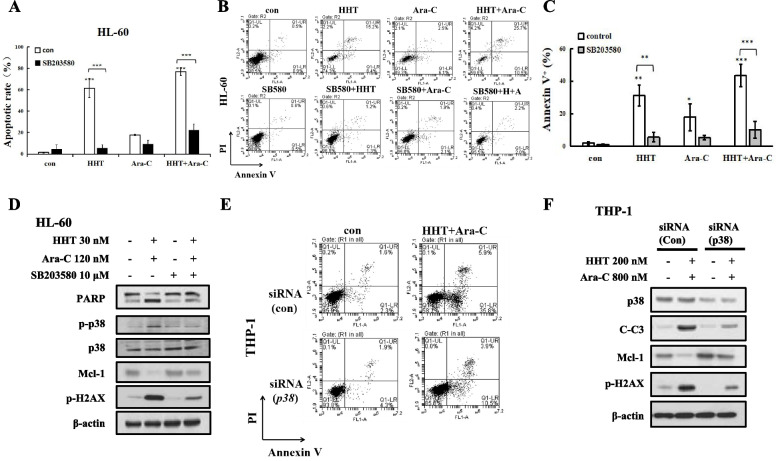
HHT enhanced Ara-C-induced apoptosis by p38 MAPK/H2AX/Mcl-1 axis. **A**, HL-60 cells were pretreated with the p38 inhibitor SB203580 (10 μM) for 4 h and then treated with 30 nM HHT and/or 120 nM Ara-C for another 24 h, after which apoptosis was examined by AO-EB staining. **B**, **C**, Apoptotic cells were detected by Annexin V/PI staining. *, *P* < 0.05; **, *P* < 0.01; ***, *P* < 0.001. **D**, Protein expression of HL-60 cells treated with SB203580, HHT or Ara-C. **E**, **F**, p38 was silenced with *p38* siRNA in THP-1 cells that were subsequently treated with HHT and Ara-C

We tested the role of p38 MAPK in HHT plus Ara-C-induced apoptosis by using p38 inhibitor SB203580 in HL-60 cells (Fig. 5A). We found that SB203580 blocked the induction of apoptosis by HHT plus Ara-C in HL-60 cells by Annexin V/PI staining (Fig. 5B, C). Compared with single agent treatment, treatment with 30 nM HHT plus 120 nM Ara-C increased apoptosis in HL-60 cells, and SB203580 also blocked HHT- and/or Ara-C-induced apoptosis. SB203580 markedly downregulated the p38 activation induced by HHT plus Ara-C, reversing PARP cleavage, Mcl-1 downregulation and H2AX phosphorylation in HL-60 cells (Fig. 5D). To confirm the role of p38 MAPK in apoptosis induction, we silenced *p38* using siRNA. Silencing *p38* attenuated HHT plus Ara-C-induced apoptosis in THP-1 cells, as determined by Annexin V/PI staining (Fig. 5E). *p38* silencing reversed the decrease in the Mcl-1 level and DNA damage and hindered the apoptosis of THP-1 cells induced by HHT and Ara-C (Fig. 5F). These results indicated that HHT enhanced Ara-C-mediated p38 and H2AX phosphorylation and Mcl-1 reduction and that the p38 MAPK/H2AX/Mcl-1 axis plays a critical regulatory role in HHT plus Ara-C-induced apoptosis in AML cells.

### HHT and Ara-C have enhanced antileukaemic effects on THP-1 xenografts

**Fig. 6:**
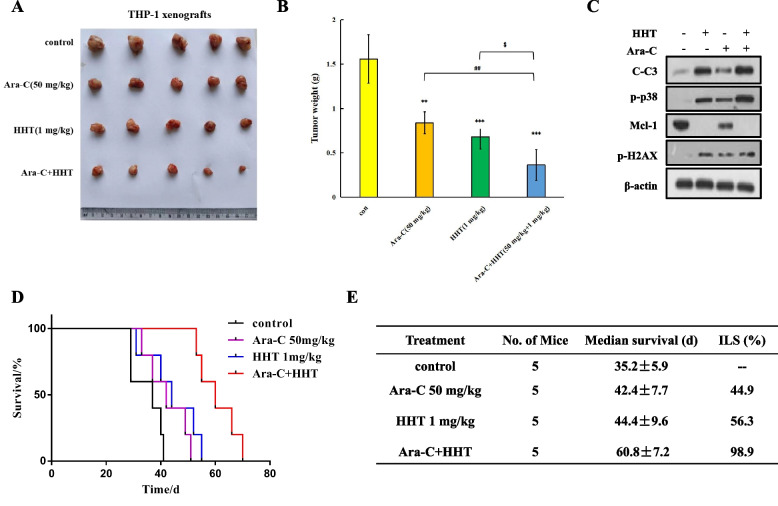
HHT in combination with **Ara-C** improved the survival of THP-1 xenografts. **A**, **B**, Tumour images and weights of 20 NOD-SCID mice subcutaneously inoculated with THP-1 cells and treated with Ara-C, HHT, or the combination treatment for 7 days. **, *P* < 0.01, ***, *P* < 0.001 compared with the control group. ^##^, *P* < 0.01 compared with the Ara-C group. ^$^, *P* < 0.05 compared with the HHT group. **C**, Protein analysis of tumour tissue. **D**, Survival curves of THP-1 xenografted mice inoculated through the tail vein and treated with 1 mg/kg HHT for 14 d and 50 mg/kg Ara-C for 7 d. **E**, Median survival and ILS of these drugs were calculated. ILS, an increase in lifespan over the control

We tested the synergistic antileukaemic activity of HHT and Ara-C in vivo. THP-1 cells were inoculated subcutaneously and treated with Ara-C (50 mg/kg), HHT (1 mg/kg), or HHT in combination with Ara-C for 7 consecutive days. The tumour growth inhibition rates resulting from Ara-C or HHT alone were 27.4 and 41.2%, respectively, and that resulting from the combination treatment was 68.6% (Fig. 6A, B). HHT in combination with Ara-C increased caspase-3 cleavage, p38 and H2AX activation and Mcl-1 downregulation (Fig. 6C). NOD-SCID mice inoculated with THP-1 cells through the tail vein were treated with Ara-C (50 mg/kg), HHT (1 mg/kg) or HHT in combination with Ara-C. The median survival time of the control group was 35.2 d. Ara-C or HHT alone improved the survival time to 42.4 d and 44.4 d, respectively. Treatment with Ara-C plus HHT prolonged survival to 60.8 days (Fig. 6D). The ILS of Ara-C or HHT alone were 44.9 and 56.3%, respectively, and their combination increased the ILS to 98.9% (Fig. 6E). There was no significant change in the weight of the mice during the treatment (Fig. S4A). These data indicated that this combination can improve the therapeutic efficacy of HHT or Ara-C to prolong the survival of patients with AML xenografts.

## Discussion

The resistance of AML cells to cytarabine is associated with the Mcl-1 protein [14]. We found that low concentrations of Ara-C inactivated p38 MAPK and blocked cell apoptosis by stabilizing Mcl-1. HHT reduced Mcl-1 levels and enhanced Ara-C-induced apoptosis via the p38 MAPK/H2AX/Mcl-1 axis.

Mcl-1 is an antiapoptotic protein of the Bcl-2 family that suppresses apoptosis [36]. Mcl-1 plays a more important role than the Bcl-2 protein in AML treatment [12]. Mcl-1 inhibitors have not been clinically developed as therapeutic drugs due to their low selectivity and cardiotoxicity [37, 38]. A selective reduction in the Mcl-1 level is a better approach for improving cytarabine therapy. Mcl-1 stabilization is regulated by the upstream MAPK pathway, which includes p38, JNK, and ERK kinases. p38 MAPK and JNK MAPK phosphorylation induced the activation of caspase-3 [39], and ERK MAPK downregulation increased apoptosis [40]. Ara-C reduced p38 phosphorylation, maintained Mcl-1 levels and caused weak DNA damage without inducing apoptosis in HL-60 and THP-1 cells (Fig. 1B, D).

Clinically, homoharringtonine plus cytarabine, which has less toxic effects, was equally effective as Dau plus Ara-C [11]. HHT is a protein translation inhibitor used for the treatment of AML and CML patients [41]. AML cell lines are sensitive to HHT, which downregulates Mcl-1 and induces apoptosis [18]. Mcl-1 binds Bim/Bak, and Bcl-2 binds Bim/Bax to block cell apoptosis [14]. Small molecule inhibitors of Bcl-2 family induce apoptosis by releasing BH3-only protein Bim from Bcl-2 proteins [14]. However, HHT does not change Bcl-2 and reduces Bim protein, unrelated to cell apoptosis induction (Fig. S2B) [30]. We found that HHT changed MAPK kinase activity without affecting Mcl-1 and apoptosis in sensitive HL-60 cells and insensitive THP-1 cells (Fig. 2C, D, G, H). The p38 inhibitor and *p38* siRNA reversed HHT-induced apoptosis (Fig. 3A-G), and the JNK and ERK inhibitors kept apoptosis (Fig. 3A, D). p38 MAPK activation reduced Mcl-1 protein levels [25, 42]. HHT enhanced apoptosis through p38 activation-mediated Mcl-1 downregulation. HHT accelerated Ara-C-mediated apoptosis induction via p38 phosphorylation and Mcl-1 downregulation (Fig. 4D, E), and the p38 inhibitor and *p38* siRNA reversed this increase in apoptosis (Fig. 5D, F). These data suggested that p38/Mcl-1 plays an important role in combination therapy.

**Fig. 7:**
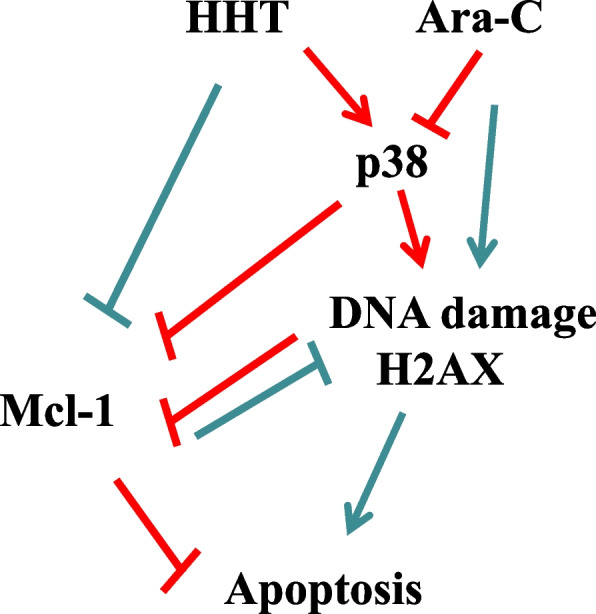
Proposed mechanism of HHT in combination with Ara-C. HHT downregulated Mcl-1 and induced DNA damage and apoptosis through the activation of p38 MAPK. HHT accelerated the Ara-C-induced increase in p38 phosphorylation and the decrease in Mcl-1 in AML cells. Response of Mcl-1 to DNA repair and DNA damage

In addition to the traditional apoptotic pathway, the Bcl-2 and Mcl-1 proteins are involved in DNA damage responses, including cell cycle checkpoint regulation and DNA repair [43, 44]. H2AX phosphorylation, a marker for DNA damage is closely related to apoptosis [45, 46]. We found that cytarabine increased H2AX phosphorylation without inducing Mcl-1 downregulation or apoptosis (Fig. 1B, D). Phosphorylated γ-H2AX and p38 MAPK proteins are located in apoptotic bodies [22, 23]. Apoptosis is related to p38-regulated H2AX phosphorylation [47, 48]. We found that Ara-C inactivated p38 MAPK (Fig. 1B, D), which is not related to DNA damage. It was reported that Ara-C increased γ-H2AX with DNA replication checkpoint kinase 1 (Chk1) phosphorylation [14]. The p38 MAPK kinase pathway is activated in response to cellular stress stimuli such as DNA damage [49]. We and others have shown that Ara-C-induced DNA double-strand breaks precede decreased Mcl-1 levels, nuclear accumulation of Mcl-1, and cell apoptosis [50]. In HL-60 cells, Ara-C activated p38 MAPK, reduced Mcl-1 levels and induced PARP and caspase-3 cleavage at higher concentrations, probably due to DNA damage induction (Fig. 4D, E). HHT enhanced Ara-C-induced apoptosis by enhancing Ara-C-induced DNA damage. We found that HHT activated p38 MAPK before H2AX phosphorylation, resulting in the downregulation of Mcl-1 expression (Fig. 2C). HHT induced apoptosis through the p38 MAPK/H2AX/Mcl-1 axis (Fig. 7). Treatment of HL-60 and THP-1 cells with the p38 inhibitor and *p38* siRNA partially attenuated DNA damage induced by HHT alone or by HHT plus Ara-C due to the presence of other mechanisms (Fig. 3E, G). Emerging evidence suggests that Mcl-1 plays an important role in DNA repair and the DNA damage response [51, 52]. HHT induced apoptosis by regulating the p38/Mcl-1 pathway to induce DNA damage (Fig. 7). The p38 MAPK/H2AX/Mcl-1 axis plays an important role in the ability of HHT to enhance Ara-C-induced apoptosis.

We tested the combined efficacy and analysed the mechanism using the optimal ratio and dosage. Traditionally, a combination regimen needs to be established by prioritizing the dosage of one drug and then increasing the concentration of another drug [31]. This method has been considered inappropriate because it ignores the proportional dependence of combination drugs [32]. The importance of drug ratios for combinations was particularly noteworthy, especially in the application of drug dosages. The synergistic drug ratio of cytarabine:daunorubicin in leukaemia cells was 5:1 for CPX-351 [53]. Using multiple AML cell lines, we found that HHT and Ara-C had synergistic effects on growth inhibition, and the optimal combination ratio was 1:4 (M/M) (Fig. 3A). HHT plus Ara-C significantly inhibited leukaemia cell growth in xenografts (Fig. 5A-D). CPX-351, as a liposomal formulation, controls drug dosage [54, 55], and a suitable carrier may provide a platform for the 1:4 release of HHT and Ara-C for optimal treatment.

HHT can directly inhibit protein synthesis or activate p38/H2AX to reduce Mcl-1-induced cell apoptosis (Fig. 7). HHT induces cell apoptosis by activating p38 MAPK to cause DNA damage (Fig. 7). Further exploration of the role of Mcl-1 in DNA repair and the DNA damage response is needed. HHT accelerated the Ara-C-induced activation of p38 and DNA damage, reduced the level of Mcl-1, induced apoptosis, and hindered the survival and proliferation of AML cells (Fig. 7). Our work highlights the importance of the p38 MAPK/H2AX/Mcl-1 axis in HHT treatment and provides a rationale for clinical HHT and Ara-C treatment for AML patients.

### Supplementary Information

**Supplementary Material 1.**

## Data Availability

The datasets used and/or analysed during the current study available from the corresponding author on reasonable request.
