# Supplementary material for: Homoharringtonine enhances cytarabine-induced apoptosis in acute myeloid leukaemia by regulating the p38 MAPK/H2AX/Mcl-1 axis
Source: BMC Cancer. 2024 Apr 24;24:520. doi: 10.1186/s12885-024-12286-7 (PMC11044605; doi:10.1186/s12885-024-12286-7)
Supplement: Supplementary file 1 — Supplementary Material 1. [file 12885_2024_12286_MOESM1_ESM.zip › Supplementary/editing certificate.pdf]

Editing Certificate

This document certifies that the manuscript

Homoharringtonine enhances cytarabine induced apoptosis in acute myeloid leukemia by regulating p38 MAPK/H2AX/Mcl-1 axis

prepared by the authors

Yang Qiu

was edited for proper English language, grammar, punctuation, spelling, and overall style by one or more of the highly qualified native English speaking editors at AJE.

This certificate was issued on **February 22, 2024** and may be verified on the AJE website using the verification code **8939-997B-999D-C094-9A2F**.

Neither the research content nor the authors' intentions were altered in any way during the editing process. Documents receiving this certification should be English-ready for publication; however, the author has the ability to accept or reject our suggestions and changes. To verify the final AJE edited version, please visit our verification page at aje.com/certificate. If you have any questions or concerns about this edited document, please contact AJE at support@aje.com.

AJE provides a range of editing, translation, and manuscript services for researchers and publishers around the world.
For more information about our company, services, and partner discounts, please visit aje.com.
