# Supplementary material for: Homoharringtonine enhances cytarabine-induced apoptosis in acute myeloid leukaemia by regulating the p38 MAPK/H2AX/Mcl-1 axis
Source: BMC Cancer. 2024 Apr 24;24:520. doi: 10.1186/s12885-024-12286-7 (PMC11044605; doi:10.1186/s12885-024-12286-7)
Supplement: Supplementary file 1 — Supplementary Material 1. [file 12885_2024_12286_MOESM1_ESM.zip › Supplementary/Supplementary Dataset File-2.pdf]

B

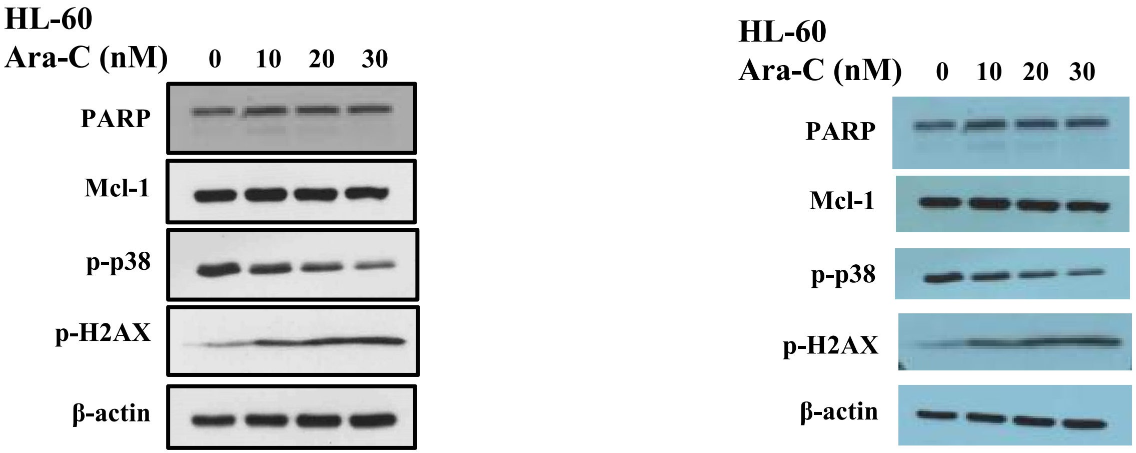
HL-60
Ara-C (nM) 0 10 20 30
PARP
Mcl-1
p-p38
p-H2AX
β-actin
HL-60
Ara-C (nM) 0 10 20 30
PARP
Mcl-1
p-p38
p-H2AX
β-actin

D

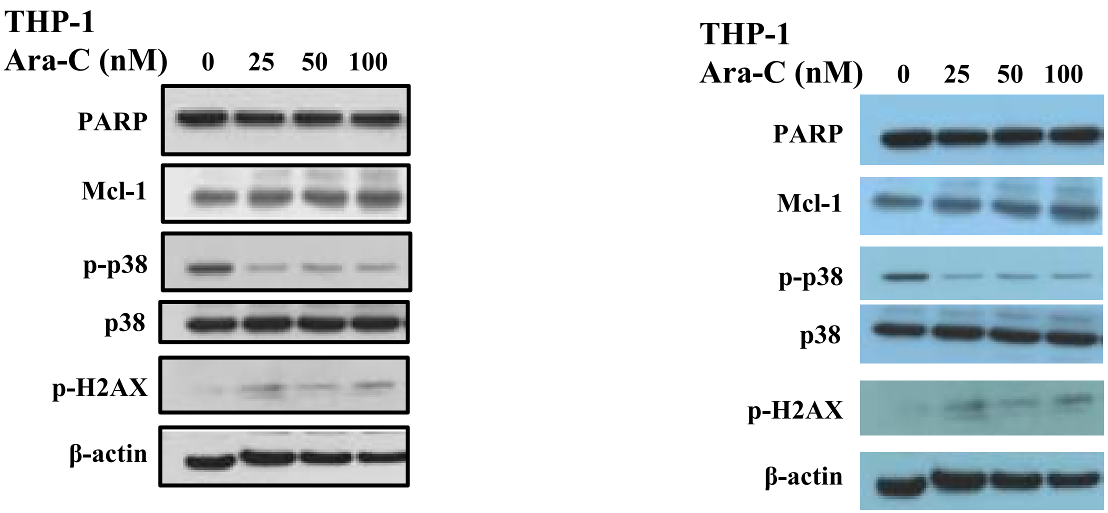
THP-1
Ara-C (nM) 0 25 50 100
PARP
Mcl-1
p-p38
p38
p-H2AX
β-actin
THP-1
Ara-C (nM) 0 25 50 100
PARP
Mcl-1
p-p38
p38
p-H2AX
β-actin

Fig.1

C

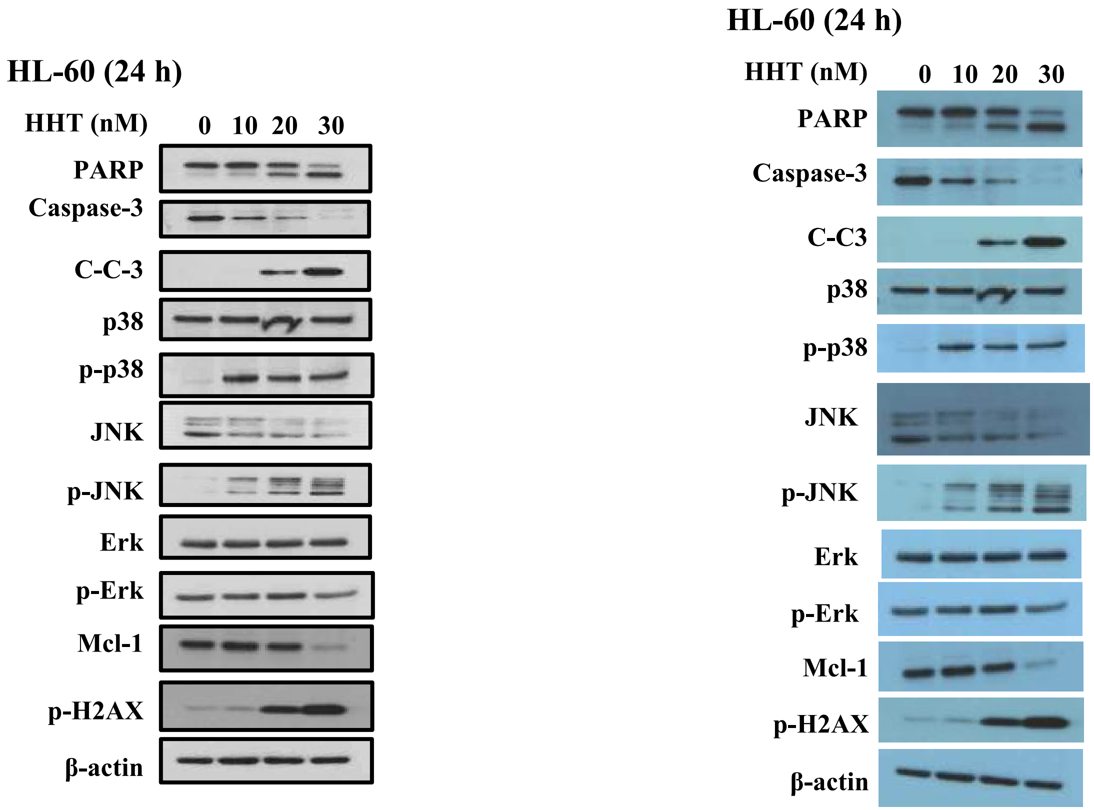
HL-60 (24 h)
HHT (nM) 0 10 20 30
PARP
Caspase-3
C-C-3
p38
p-p38
JNK
p-JNK
Erk
p-Erk
Mcl-1
p-H2AX
β-actin
HL-60 (24 h)
HHT (nM) 0 10 20 30
PARP
Caspase-3
C-C3
p38
p-p38
JNK
p-JNK
Erk
p-Erk
Mcl-1
p-H2AX
β-actin

D

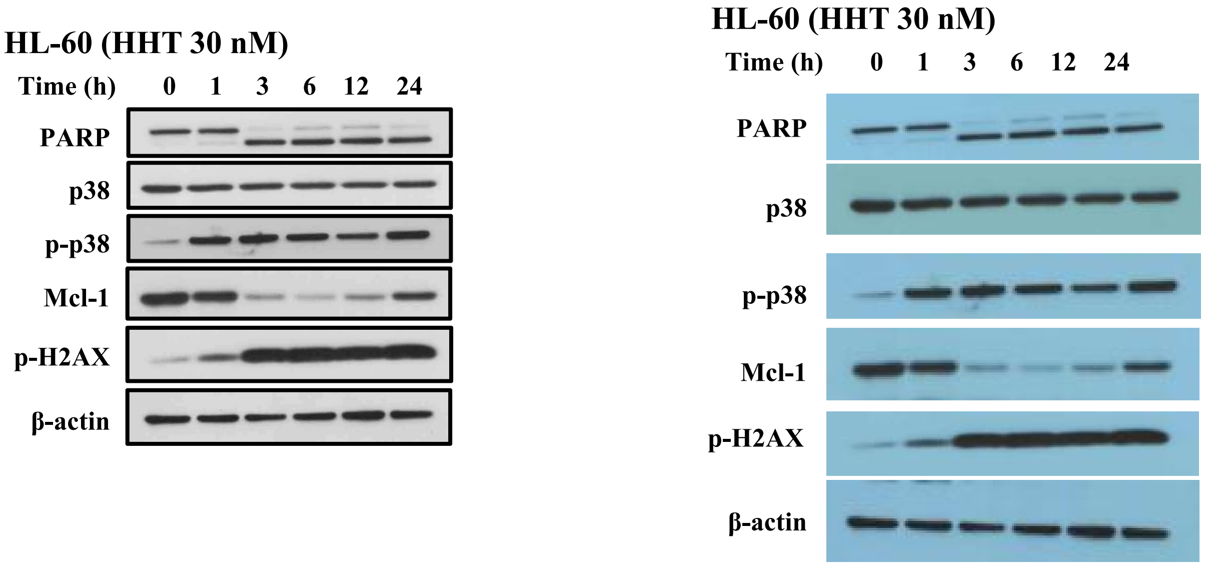
HL-60 (HHT 30 nM)
Time (h) 0 1 3 6 12 24
PARP
p38
p-p38
Mcl-1
p-H2AX
β-actin
HL-60 (HHT 30 nM)
Time (h) 0 1 3 6 12 24
PARP
p38
p-p38
Mcl-1
p-H2AX
β-actin

Fig.2

G

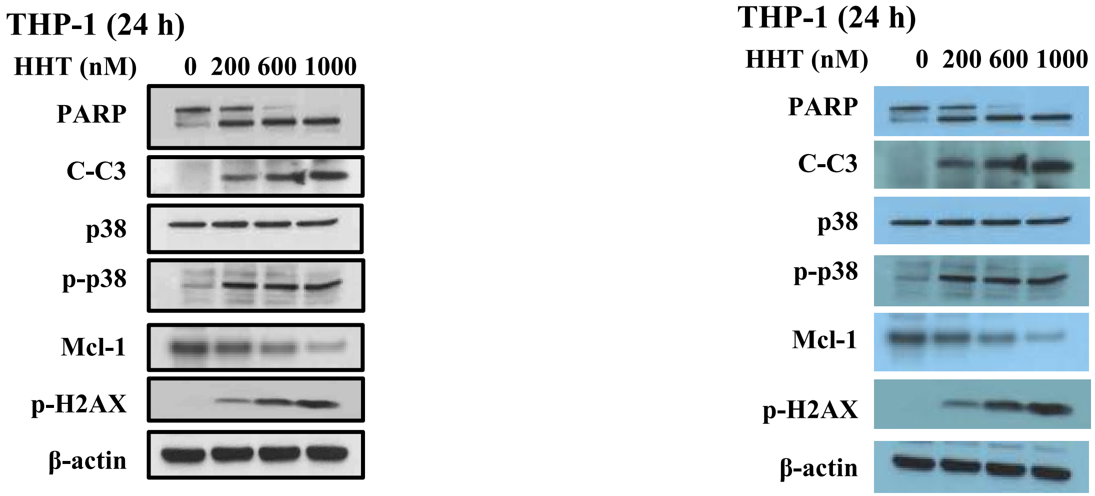
THP-1 (24 h)
HHT (nM) 0 200 600 1000
PARP
C-C3
p38
p-p38
Mcl-1
p-H2AX
β-actin
THP-1 (24 h)
HHT (nM) 0 200 600 1000
PARP
C-C3
p38
p-p38
Mcl-1
p-H2AX
β-actin

H

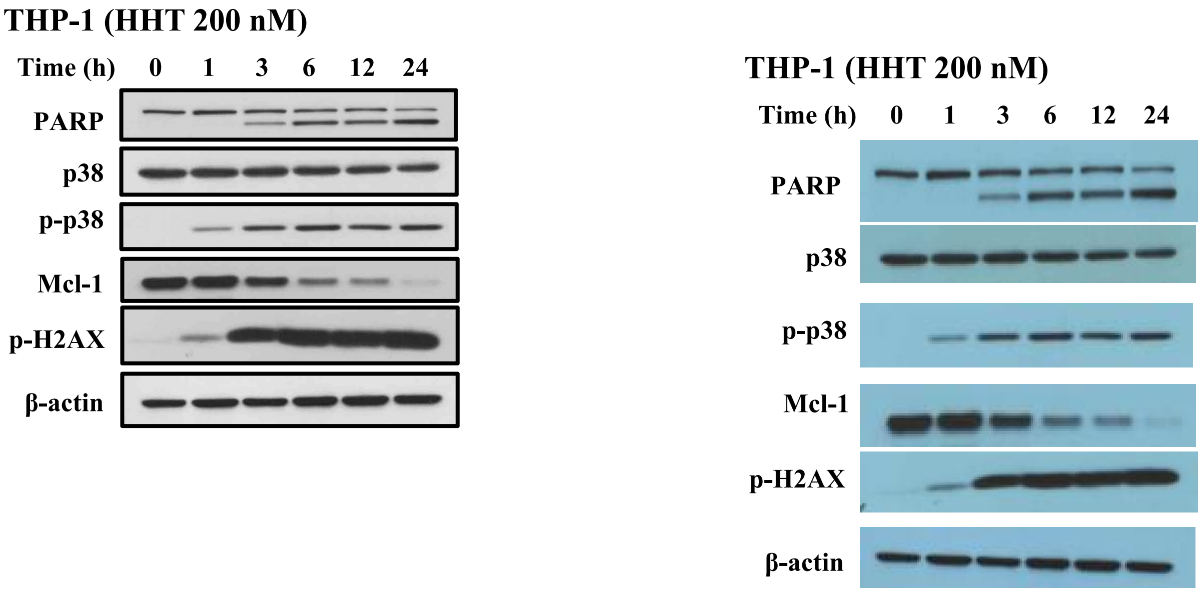
THP-1 (HHT 200 nM)
Time (h) 0 1 3 6 12 24
PARP
p38
p-p38
Mcl-1
p-H2AX
β-actin
THP-1 (HHT 200 nM)
Time (h) 0 1 3 6 12 24
PARP
p38
p-p38
Mcl-1
p-H2AX
β-actin

Fig.2

C

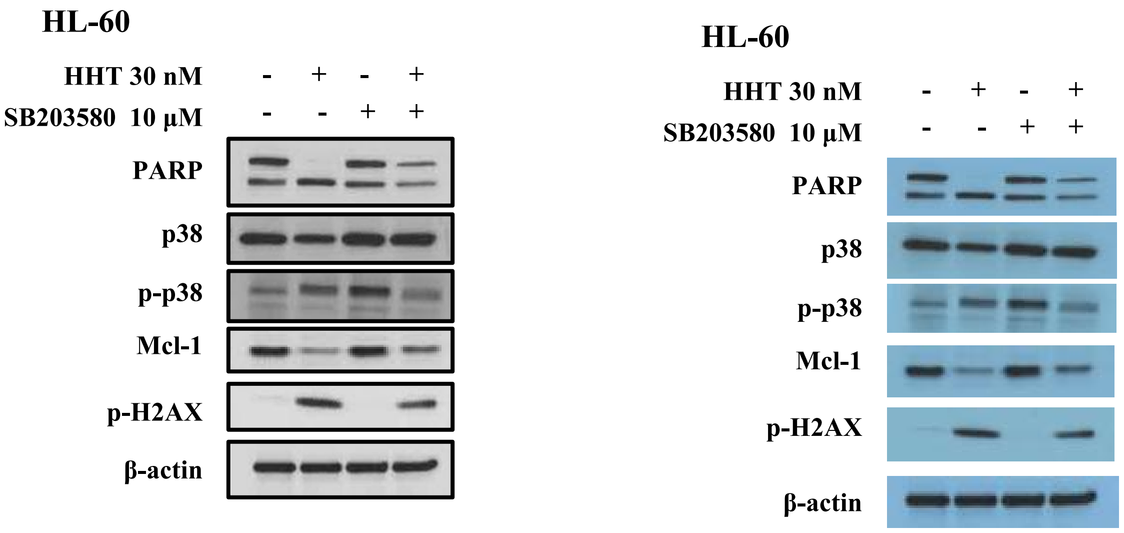
HL-60
HHT 30 nM
SB203580 10 μM
PARP
p38
p-p38
Mcl-1
p-H2AX
β-actin
HL-60
HHT 30 nM
SB203580 10 μM
PARP
p38
p-p38
Mcl-1
p-H2AX
β-actin

E

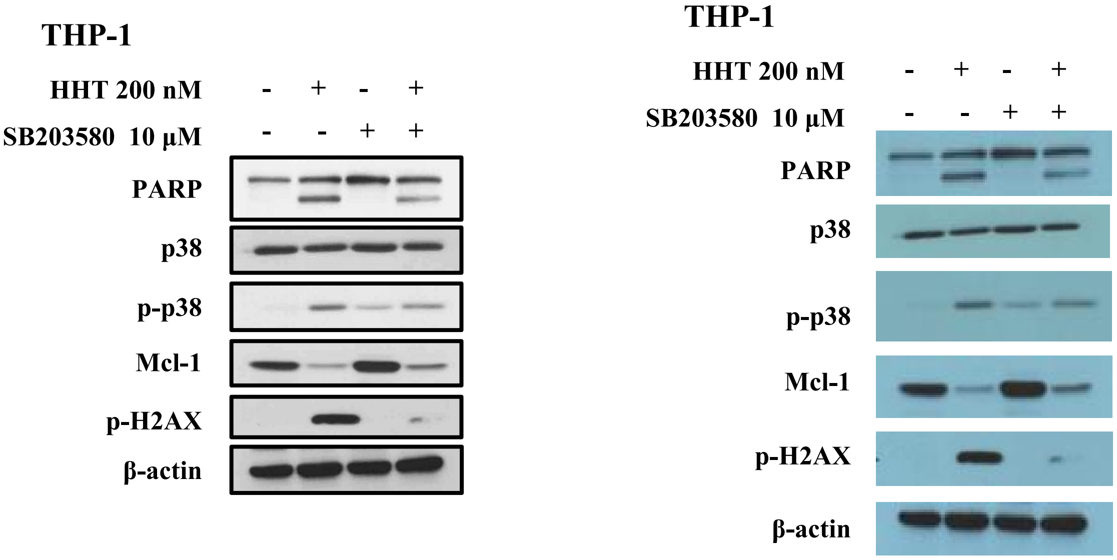
THP-1
HHT 200 nM
SB203580 10 μM
PARP
p38
p-p38
Mcl-1
p-H2AX
β-actin
THP-1
HHT 200 nM
SB203580 10 μM
PARP
p38
p-p38
Mcl-1
p-H2AX
β-actin

Fig.3

G

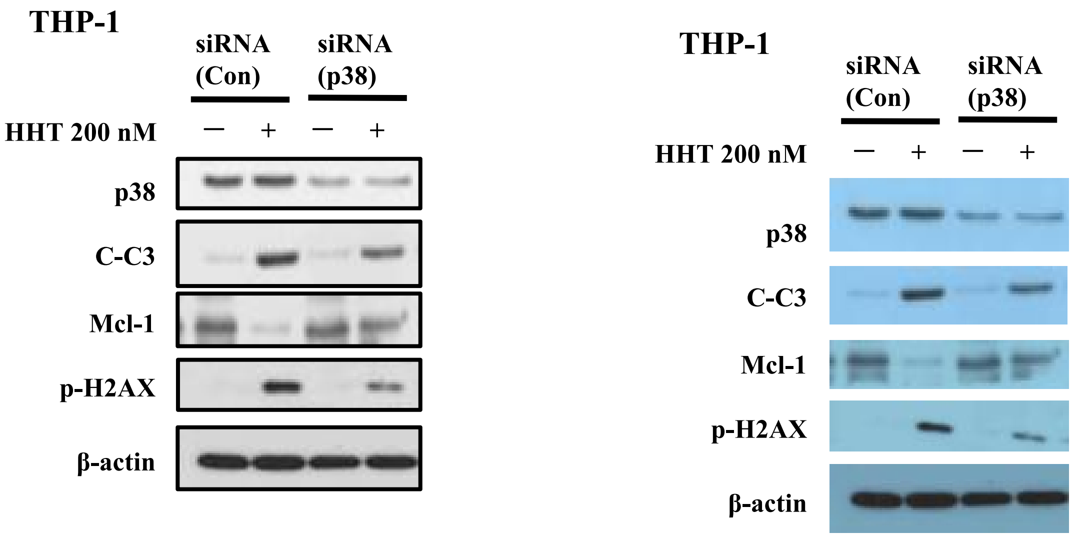
THP-1
siRNA (Con)
siRNA (p38)
HHT 200 nM
— + — +
p38
C-C3
Mcl-1
p-H2AX
β-actin
THP-1
siRNA (Con)
siRNA (p38)
HHT 200 nM
— + — +
p38
C-C3
Mcl-1
p-H2AX
β-actin

Fig.3

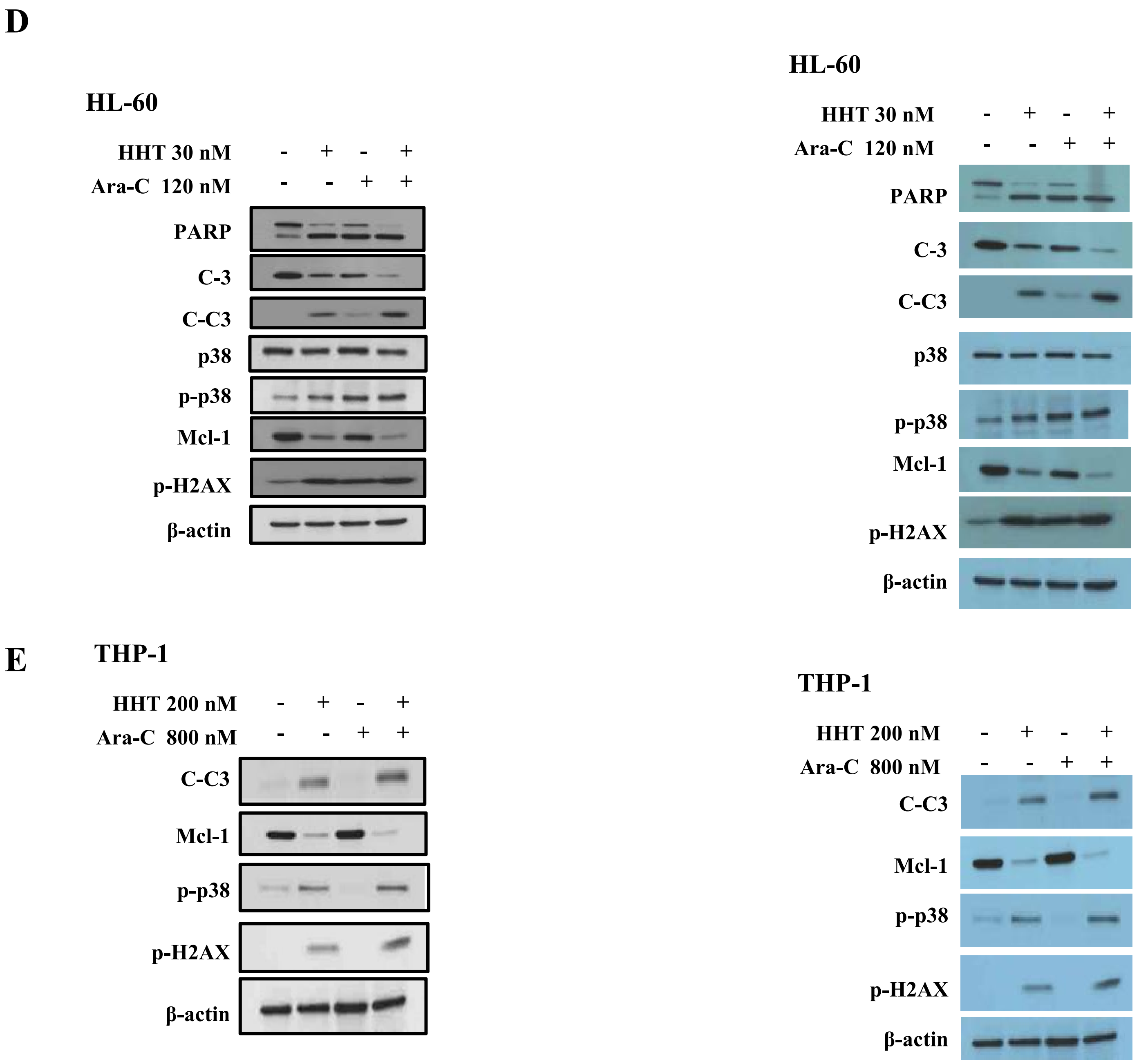
D
HL-60
HHT 30 nM - + - +
Ara-C 120 nM - - + +
PARP
C-3
C-C3
p38
p-p38
Mcl-1
p-H2AX
β-actin
HL-60
HHT 30 nM - + - +
Ara-C 120 nM - - + +
PARP
C-3
C-C3
p38
p-p38
Mcl-1
p-H2AX
β-actin
E
THP-1
HHT 200 nM - + - +
Ara-C 800 nM - - + +
C-C3
Mcl-1
p-p38
p-H2AX
β-actin
THP-1
HHT 200 nM - + - +
Ara-C 800 nM - - + +
C-C3
Mcl-1
p-p38
p-H2AX
β-actin

Fig.4

D

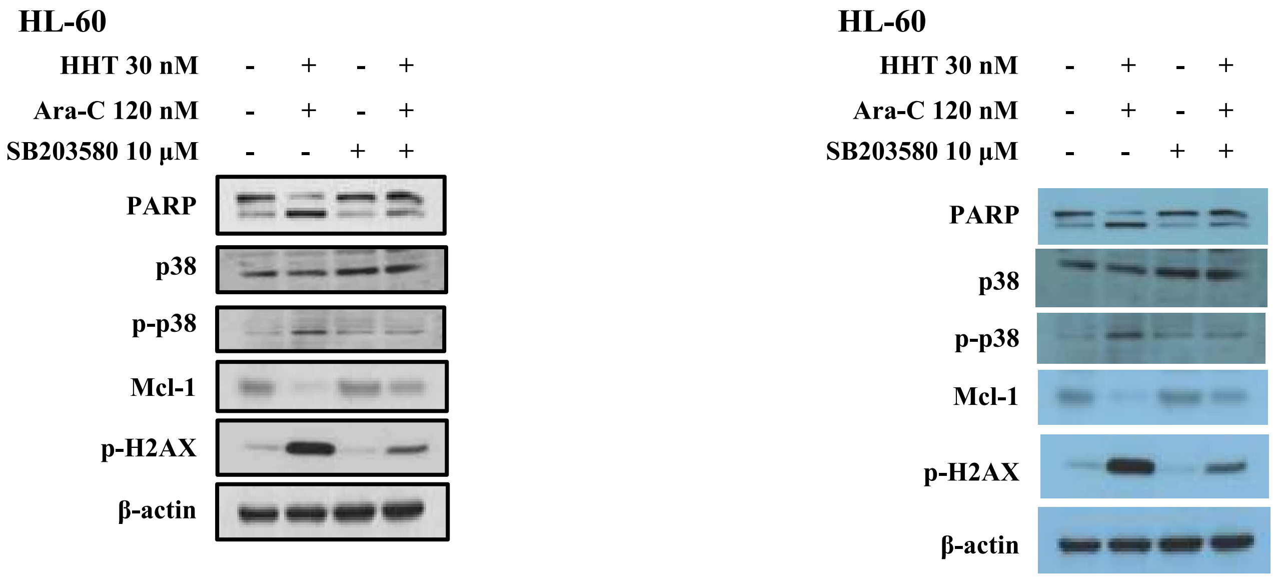
HL-60
HHT 30 nM - + - +
Ara-C 120 nM - + - +
SB203580 10 µM - - + +
PARP
p38
p-p38
Mcl-1
p-H2AX
β-actin
HL-60
HHT 30 nM - + - +
Ara-C 120 nM - + - +
SB203580 10 µM - - + +
PARP
p38
p-p38
Mcl-1
p-H2AX
β-actin

F

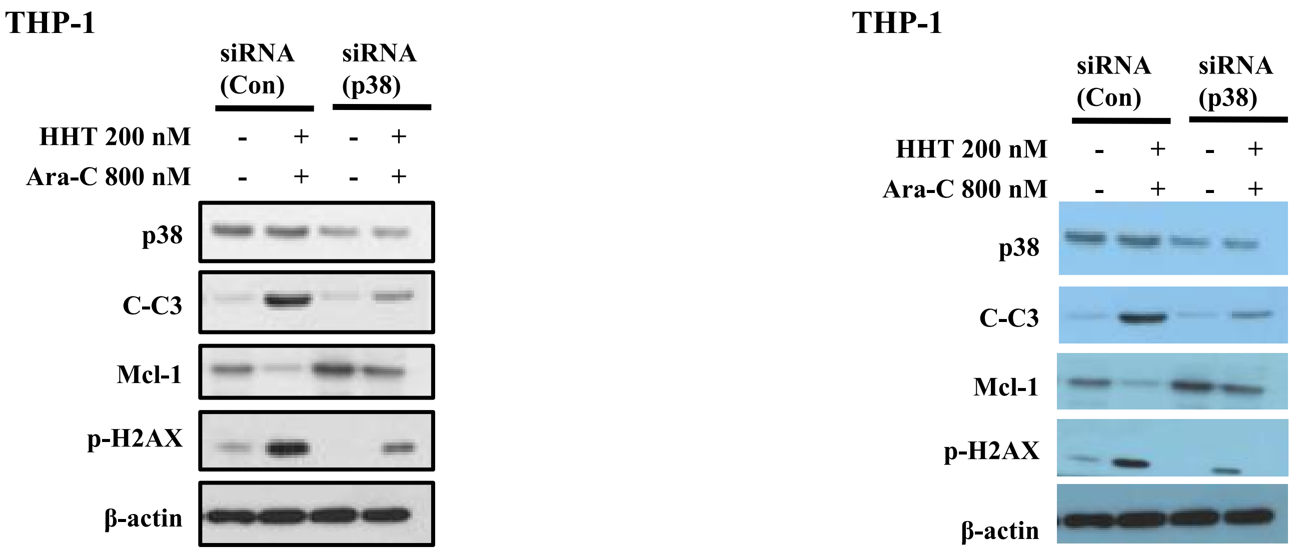
THP-1
siRNA (Con)
siRNA (p38)
HHT 200 nM - + - +
Ara-C 800 nM - + - +
p38
C-C3
Mcl-1
p-H2AX
β-actin
THP-1
siRNA (Con)
siRNA (p38)
HHT 200 nM - + - +
Ara-C 800 nM - + - +
p38
C-C3
Mcl-1
p-H2AX
β-actin

Fig.5

C

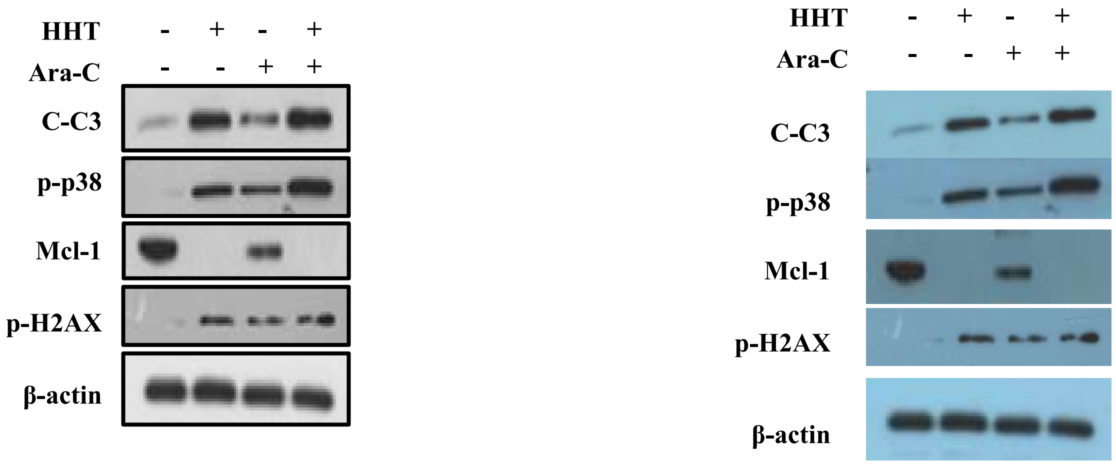
HHT - + - +
Ara-C - - + +
C-C3
p-p38
Mcl-1
p-H2AX
β-actin
HHT - + - +
Ara-C - - + +
C-C3
p-p38
Mcl-1
p-H2AX
β-actin

Fig.6

A

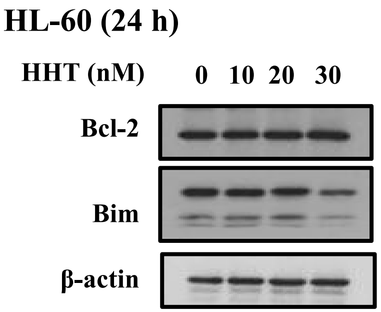
HL-60 (24 h)
HHT (nM)
0 10 20 30
Bcl-2
Bim
β-actin

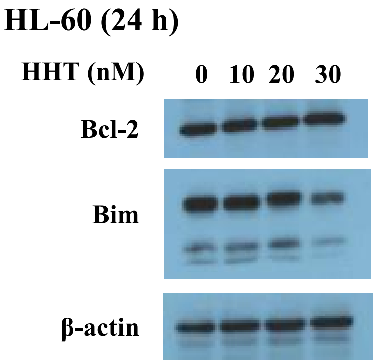
HL-60 (24 h)
HHT (nM)
0 10 20 30
Bcl-2
Bim
β-actin

Fig.S1
