# Supplementary figures and images for: Homoharringtonine enhances cytarabine-induced apoptosis in acute myeloid leukaemia by regulating the p38 MAPK/H2AX/Mcl-1 axis
Source: BMC Cancer. 2024 Apr 24;24:520. doi: 10.1186/s12885-024-12286-7 (PMC11044605; doi:10.1186/s12885-024-12286-7)

A

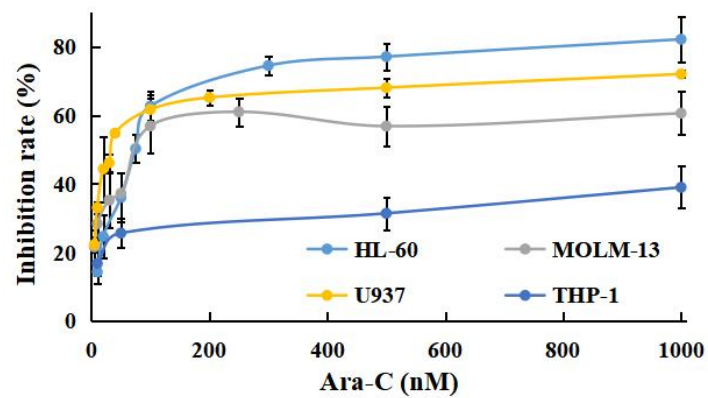

Fig.S1

**A**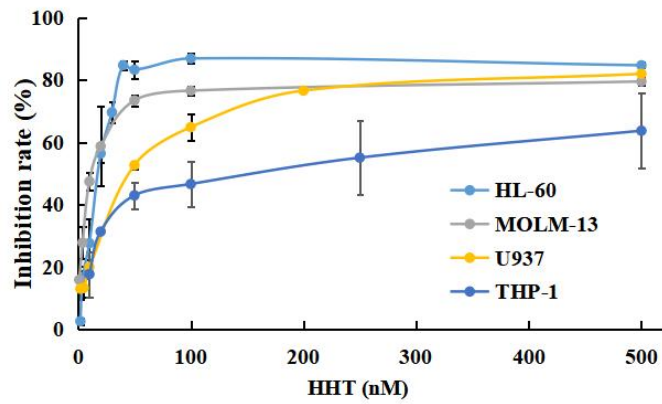**B****HL-60 (24 h)**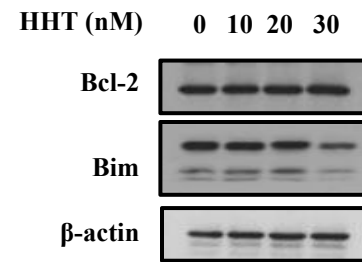**Fig.S2**

**A**

| Cell lines | GI <sub>50</sub> (nM) |                   |
|------------|-----------------------|-------------------|
|            | HHT                   | Ara-C             |
| HL-60      | 16.07 ± 2.60          | 77.87 ± 8.67      |
| MOLM-13    | 13.83 ± 1.91          | 140.80 ± 17.22    |
| U937       | 48.77 ± 3.24          | 47.58 ± 3.05      |
| THP-1      | 213.23 ± 43.55        | 7155.09 ± 1184.26 |

**B**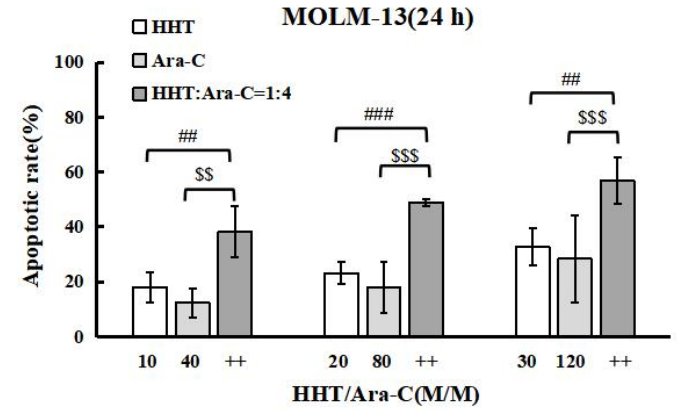**C**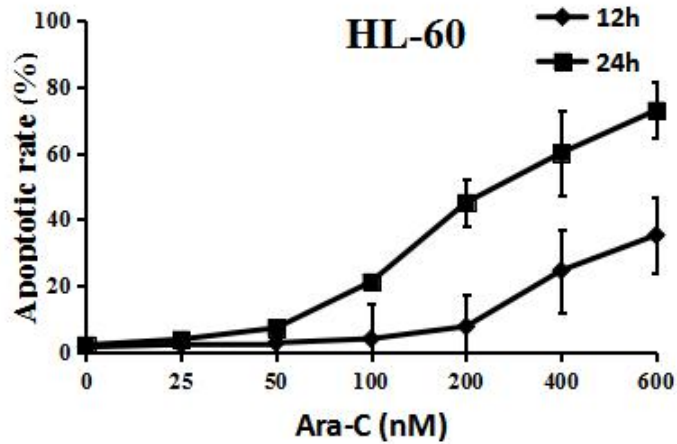

A

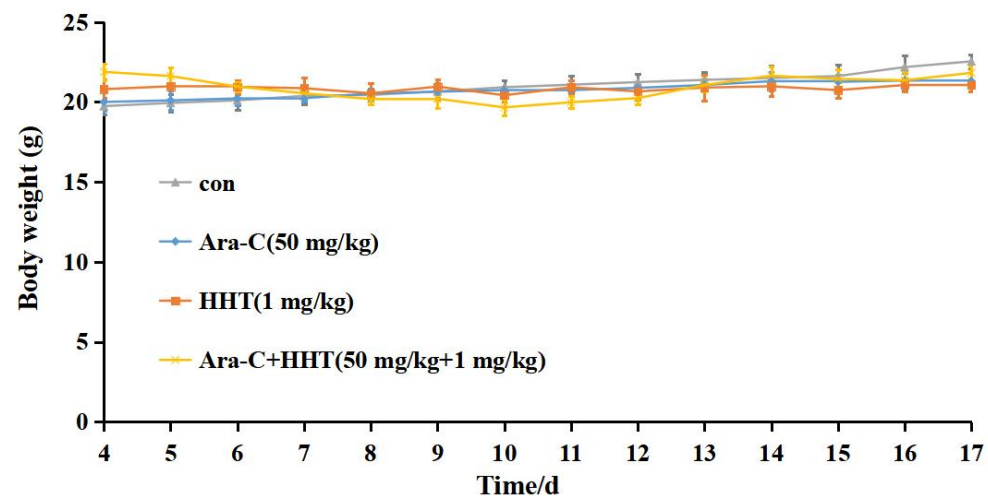

Fig.S4

Supplement: Supplementary file 1 — Supplementary Material 1. [file 12885_2024_12286_MOESM1_ESM.zip › Supplementary/Supplementary figure File-2.pdf]
