# Supplementary material for: Homoharringtonine enhances cytarabine-induced apoptosis in acute myeloid leukaemia by regulating the p38 MAPK/H2AX/Mcl-1 axis
Source: BMC Cancer. 2024 Apr 24;24:520. doi: 10.1186/s12885-024-12286-7 (PMC11044605; doi:10.1186/s12885-024-12286-7)
Supplement: Supplementary file 1 — Supplementary Material 1. [file 12885_2024_12286_MOESM1_ESM.zip › Supplementary/supplementary file.doc]

**Supplementary** Fig. 1 Anti-leukaemia effect of Ara-C. **A,** The rate of cell growth inhibition induced by Ara-C for 24 h in HL-60, THP-1, MOLM-13 and U937 cells, as determined by trypan blue staining.

**Supplementary** Fig. 2 Anti-leukaemia effect of HHT. **A,** The rate of cell growth inhibition induced by HHT for 24 h in HL-60, THP-1, MOLM-13 and U937 cells, as determined by trypan blue staining. **B,** Protein regulation in HL-60 cells treated with increasing concentrations of HHT for 24 h.

**Supplementary** Fig. 3 Anti-leukaemia effect of HHT and Ara-C. **A,** The GI_50_ values of HHT or Ara-C for 24 h in HL-60, THP-1, MOLM-13 and U937 cells with trypan blue exclusion were calculated. **B,** MOLM-13 cells treated with the combination at a fixed ratio of 1:4, containing 10 nM, 20 nM, 30 nM HHT and 40 nM, 80 nM, 120 nM Ara-C for 24 h were examined by AO-EB staining. ^##^, *P* < 0.01; ^###^, *P*＜0.001. ^$$^, *P* < 0.01;^$$$^, *P*＜0.001. **C,** The apoptosis of HL-60 cells treated with increasing concentrations of Ara-C for 12 h and 24 h.

**Supplementary** Fig. 4 **HHT in combination with Ara-C improved the survival of THP-1 xenografts**. A, Mouse weights during treatment.
